# Association of Common Variants in eNOS Gene with Primary Open Angle Glaucoma: A Meta-Analysis

**DOI:** 10.1155/2016/1348347

**Published:** 2016-05-08

**Authors:** Yang Xiang, Yi Dong, Xuan Li, Xin Tang

**Affiliations:** ^1^Clinical College of Ophthalmology, Tianjin Medical University, 4 Gansu Road, Tianjin 300020, China; ^2^Tianjin Eye Hospital, 4 Gansu Road, Tianjin 300020, China; ^3^Tianjin Key Laboratory of Ophthalmology and Visual Science, 4 Gansu Road, Tianjin 300020, China; ^4^Tianjin Eye Institute, 4 Gansu Road, Tianjin 300020, China

## Abstract

*Purpose*. To clarify the association of endothelial nitric oxide synthase (eNOS) polymorphisms and primary open angle glaucoma (POAG).* Methods*. After a systematic literature search in the MEDLINE, EMBASE, and ISI Web of Science databases, all relevant studies evaluating the association between the polymorphisms (rs2070744 and rs1799983) of eNOS gene and POAG were screened and included. The pooled odds ratios (ORs) and the 95% confidence interval (CI) of each single-nucleotide polymorphism (SNP) in five genetic models were estimated using fixed-effect model if *I* ^2^ < 50% in the test for heterogeneity; otherwise the random-effects model was used.* Results*. Thirty-one records were obtained, with five being suitable for meta-analysis. The overall results showed that both TT genotype in rs2070744 and GG genotype in rs1799983 are associated with decreased risk of POAG susceptibility. Stratified analysis based on ethnicity showed that the association of rs2070744 with POAG remained only in Caucasians. Results of subgroup analysis by sex indicated association between both polymorphisms and POAG in female group, but not in male group.* Conclusions*. TT genotype and/or T-allele in rs2070744, as well as GG genotype and/or G-allele in rs1799983, was associated with decreased risk for POAG overall and in female group.

## 1. Introduction

Glaucoma is a common, complex, heterogenous disease and it constitutes the major cause of irreversible blindness worldwide [1]. In 2013, the number of people (aged 40–80 years) with glaucoma was estimated to be 64.3 million, increasing to 76.0 million in 2020 and 111.8 million in 2040, disproportionally affecting people residing in Asia and Africa [2]. Primary open angle glaucoma (POAG), the most common type of glaucoma in all populations, is characterized by progressive damage of retinal ganglion cells (RGCs) and their axons, leading to the pathognomonic remodeling of the optic nerve head and subsequent irrevocable vision loss [3]. The known risk factors for POAG include a higher age, African ancestry, refractive error, and a positive family history for glaucoma, apart from elevated intraocular pressure (IOP), an established risk contributor [4–6]. Furthermore, there is growing evidence that vascular [7, 8] and genetic [9–11] components may pose a potential risk to POAG patients, including both those with normal and elevated IOP.

Nitric oxide (NO) is an active biologic agent involved in diverse physiologic processes [12]. NO generated by endothelial nitric oxide synthase (eNOS) has been found to contribute to vasodilatation, increased local blood flow, and decreased vascular resistance in ocular circulation [13, 14]. Hence, changes in the activity of eNOS determined by genetic variations and environmental factors may play an important role in the pathogenesis of glaucoma. Several studies were conducted to evaluate the association of eNOS polymorphisms with risk of POAG but presented inconsistent results [15–20]. During seven functional single-nucleotide polymorphisms (SNPs) reported in relevant studies, the controversy was mainly centered on the two most important SNPs, T-786C (rs2070744) and Glu298Asp (rs1799983). Thus the current meta-analysis aims to assess the strength of the evidence for an effect of these two polymorphisms on POAG risk by combining data from all relevant eligible studies.

## 2. Methods

### 2.1. Literature Search

A systematic literature search was conducted in the MEDLINE, EMBASE, and Web of Science databases (accessed on November 30, 2015) with the following free words and MeSH terms: “glaucoma”, “open angle”, “Endothelial nitric oxide synthase”, “eNOS”, “polymorphism(s)”, “single nucleotide polymorphism”, and “SNP”. We also supplemented our search by screening the reference lists of all the retrieved studies, as well as genome-wide association studies (GWAS) performed for glaucoma to which we have the access.

### 2.2. Inclusion and Exclusion Criteria

Eligible studies were included if they (1) evaluated the association between eNOS and POAG; (2) compared unrelated POAG cases and normal controls identified by complete ophthalmological examination in defined populations; (3) provided an odds ratio (OR) with 95% confidence interval (CI) in case and control groups, respectively, or other data which could be calculated to estimate an OR; and (4) were original research articles. All animal studies, case reports, abstracts from conferences, full-texts with incomplete data, and reviews were excluded. As for duplicate studies retrieving data from the same source, ones with available data and the largest sample size were brought into the analysis list. Although we did not define language in the review process, the articles in the final analysis were all in English.

### 2.3. Literature Review and Data Extraction

Two investigators (Yang Xiang and Yi Dong) extracted data from the retrieved records and confirmed the validity of the included articles independently. The following variables were extracted: author, year of publication, ethnicity of subjects, demographic information, the numbers of cases and controls, results of Hardy-Weinberg equilibrium (HWE) test when reported, and the allele and genotype counts or frequencies of each SNP. When the allele or genotype counts were not given specially in some articles, they were calculated from the frequencies and then rounded to the nearest integer. A final review was performed by other reviewers (Xuan Li and Xin Tang) while the discrepancy was resolved through discussion.

### 2.4. Meta-Analysis and Test for Potential Bias

The Chi-square test was utilized to check whether the genotype distribution in controls was consistent with HWE for studies that did not report relevant data. To assess the strength of association between the polymorphisms (rs2070744 and rs1799983) of eNOS gene and POAG susceptibility, we estimated crude OR with its 95% CI under allele model (T versus C, G versus A), homozygote model (TT versus CC, GG versus AA), heterozygote model (TC versus CC, GA versus AA), dominant model (TT + TC versus CC, GG + GA versus AA), and recessive model (TT versus TC + CC, GG versus GA + AA), respectively.

Interstudy heterogeneity was detected using the Chi-square-based *Q* statistic test as well as the *I* ^2^ metric. If *P*_*Q*_ ≤ 0.10 or *I* ^2^ > 50%, which indicated significant heterogeneity in the comparison models among studies [21], the pooled ORs were calculated with a random-effects model (DerSimonian and Laird method) [22]. Otherwise, the fixed-effects model was considered more appropriate (Mantel-Haenszel method) [23]. We also conducted subgroup analysis based on ethnicity, as well as sex where applicable. Publication bias was investigated by Begg's tests and Egger's linear regression test [24, 25]. The statistical analysis was done with Stata 12.0 and the values of *P* < 0.05 were considered statistically significant.

## 3. Results

### 3.1. Literature Search and Characteristics

**Figure 1:**
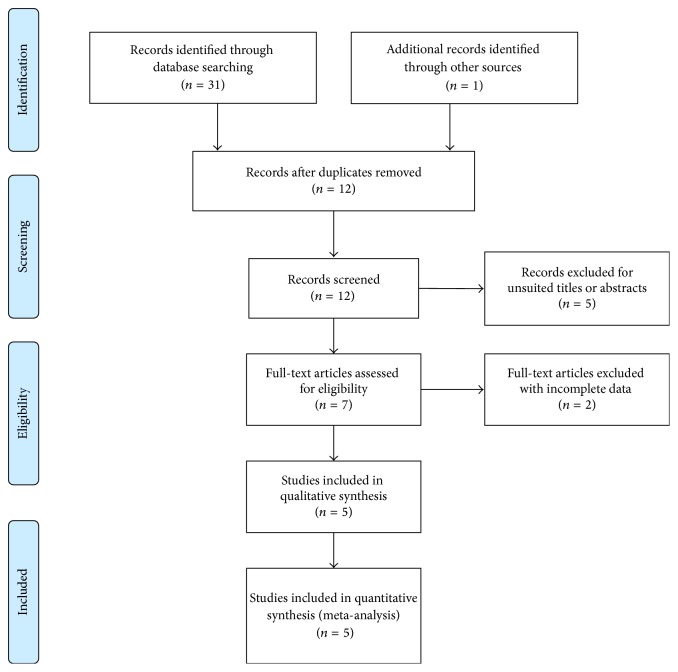
PRISMA flow diagram of studies included in the meta-analysis.

The workflow and results of the literature review are shown in Figure 1. A total of thirty-one records were initially identified for the meta-analysis. Of the thirty-one, twenty-six studies were excluded due to duplicated publications, unsuitable titles or abstracts, or incomplete data. In total, five eligible studies [15–19] were included and reviewed. Seven SNPs of eNOS gene, including rs2070744, rs1799983, rs743507, rs3793342, rs7830, rs11771443, and rs3918188, were evaluated for possible association with POAG while five SNPs, apart from rs2070744 and rs1799983, were reported in only one or two studies, the data of which were interpreted to be insufficient to perform a qualified meta-analysis. Consequently, the combined study population investigating rs2070744 (consisting of 1156 cases and 1879 controls) and rs1799983 (consisting of 1230 cases and 2035 controls) are involved in our meta-analysis, and the detailed characteristics of the included studies are listed in Tables 1 and 2.

### 3.2. Meta-Analysis Results

**Figure 2:**
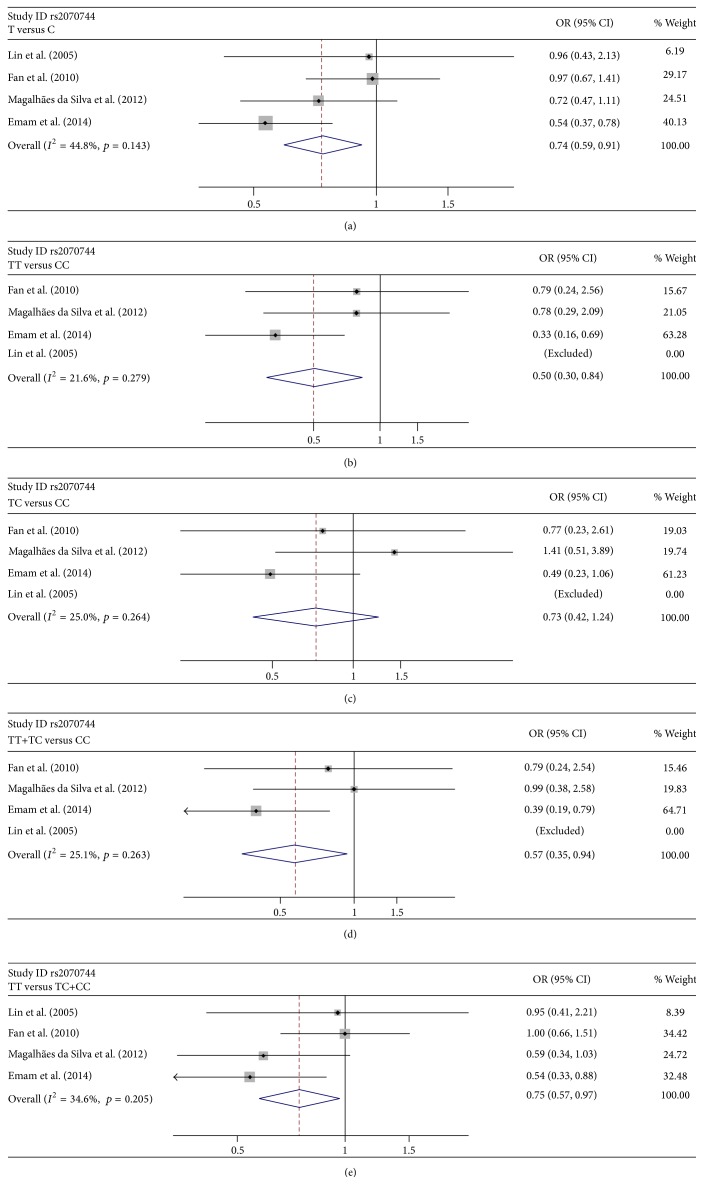
Forest plots of the association of rs2070744 with POAG. Every study was represented by a square whose size was proportional to the weight of the study. Diamond indicated summary odds ratios (ORs) with its corresponding 95% confidence interval (95% CI). (a) Forest plot for rs2070744 and POAG risk in the genetic model of T-allele versus C allele. (b) Forest plot for rs2070744 and POAG risk in the genetic model of TT versus CC. (c) Forest plot for rs2070744 and POAG risk in the genetic model of TC versus CC. (d) Forest plot for rs2070744 and POAG risk in the genetic model of TT + TC versus CC. (e) Forest plot for rs2070744 and POAG risk in the genetic model of TT versus TC + CC.

**Figure 3:**
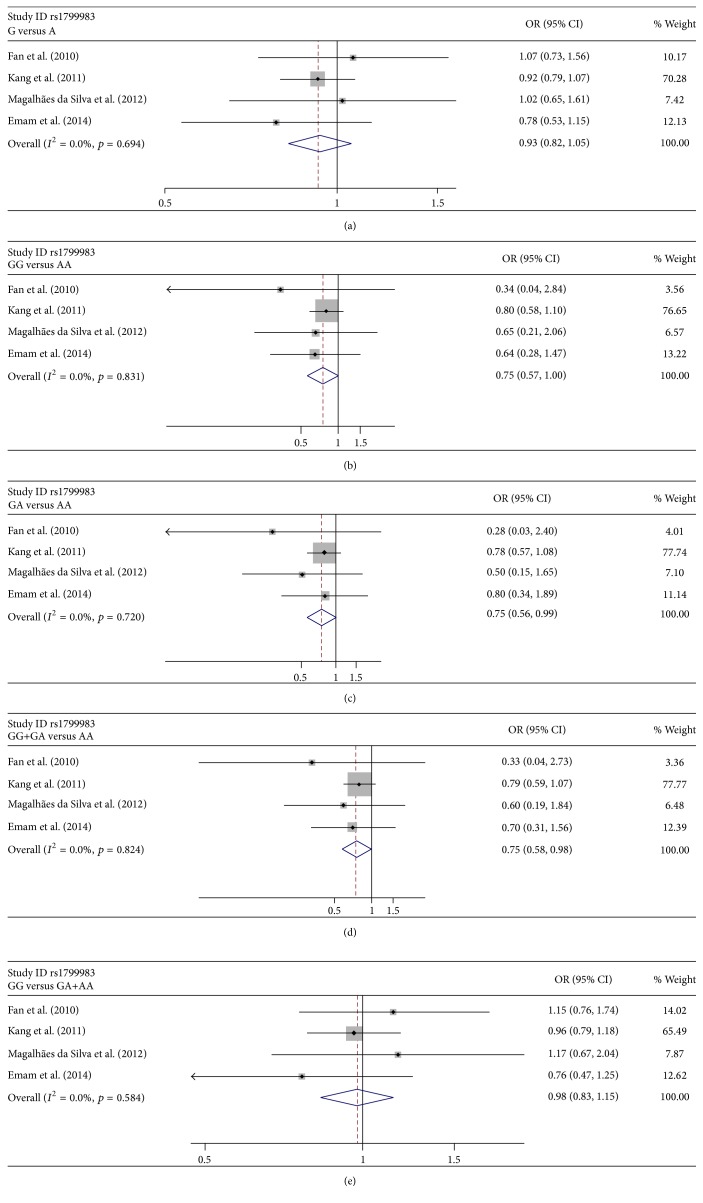
Forest plots of the association of rs1799983 with POAG. Every study was represented by a square whose size was proportional to the weight of the study. Diamond indicated summary odds ratios (ORs) with its corresponding 95% confidence interval (95% CI). (a) Forest plot for rs1799983 and POAG risk in the genetic model of G-allele versus A allele. (b) Forest plot for rs1799983 and POAG risk in the genetic model of GG versus AA. (c) Forest plot for rs1799983 and POAG risk in the genetic model of GA versus AA. (d) Forest plot for rs1799983 and POAG risk in the genetic model of GG + GA versus AA. (e) Forest plot for rs1799983 and POAG risk in the genetic model of GG versus GA + AA.

Tables 3 and 4 show the summary results for the association between eNOS rs2070744 and rs1799983 and risk for POAG. Low heterogeneity was present among all the publications involved for all the genetic models (Tables 3 and 4). Thus, the data were combined using the fixed-effects model. For rs2070744, the data was pooled from 4 sample collections without HWE deviation, and the overall results showed significant association between rs2070744 and POAG (OR = 0.736, 95% CI = 0.594–0.912 for T-allele versus C allele (Figure 2(a)); OR = 0.498, 95% CI = 0.296–0.838 for TT versus CC (Figure 2(b)); OR = 0.573, 95% CI = 0.348–0.943 for TT + CC versus CC (Figure 2(d)); OR = 0.746, 95% CI = 0.575–0.967 for TT versus TT + TC (Figure 2(e))). Statistically significant association was also observed between rs1799983 and POAG (OR = 0.753, 95% CI = 0.568–0.997 for GG versus AA (Figure 3(b)); OR = 0.745, 95% CI = 0.559–0.993 for GA versus AA (Figure 3(c)); OR = 0.752, 95% CI = 0.576–0.983 for GG + GA versus AA (Figure 3(d))).

To further explore the association, stratified analysis was performed based on ethnicity (Caucasians and Asians) and sex. For rs2070744, the results showed that the association between rs2070744 and POAG was significant in Caucasians (OR = 0.607, 95% CI = 0.460–0.803 for T-allele versus C allele; OR = 0.444, 95% CI = 0.249–0.791 for TT versus CC; OR = 0.534, 95% CI = 0.308–0.925 for TT + CC versus CC; OR = 0.563, 95% CI = 0.390–0.812 for TT versus TT + TC) but not in Asians (Table 3). Stratified analysis based on sex supported a link only in female group (OR = 0.490, 95% CI = 0.333–0.721 for T-allele versus C allele; OR = 0.268, 95% CI = 0.112–0.642 for TT versus CC; OR = 0.328, 95% CI = 0.144–0.749 for TT + CC versus CC; OR = 0.423, 95% CI = 0.260–0.689 for TT versus TT + TC (Table 3)). For rs1799983, the results revealed no evidence of the association in neither Caucasians nor Asians (Table 4). As expected, statistical association was found in female subgroup (OR = 0.665, 95% CI = 0.471–0.938 for GG versus AA; OR = 0.674, 95% CI = 0.474–0.958 for GA versus AA; OR = 0.666, 95% CI = 0.481–0.923 for GG + GA versus AA). Consistently, we still found no relationship in the male subgroup (Table 4).

### 3.3. Publication Bias

Publication bias was quantitatively assessed by Begg's tests and Egger's tests. In the overall analysis, there was no evidence of publication bias detected for rs2070744 (Table 3). With regard to rs1799983, Egger's regression test suggested a weak indication of publication bias, whereas Begg's rank correlation test did not identify evidence of substantial publication bias (Table 4).

## 4. Discussion

We reviewed a broad selection of publications found in electronic databases and performed a meta-analysis, in an attempt to identify the effects of polymorphisms of the eNOS gene on the pathogenesis of POAG. Five eligible studies were involved and available data in this regard were conflicting [15–19]. After the results were pooled, the main finding of this study is that TT genotype and/or T-allele in rs2070744, as well as GG genotype and/or G-allele in rs1799983, could protect individuals from POAG risk. Stratified analysis based on ethnicity showed that the association of rs2070744 with POAG remained only in Caucasians, while no association between rs1799983 and POAG was found in either Caucasians or Asians. To further explore the association, we performed subgroup analysis by sex. The results indicated that TT genotype and/or T-allele in rs2070744 and GG genotype and/or G-allele in rs1799983 were favorable factors for POAG in female group, but not in male group.

Generated by eNOS via the conversion of L-arginine to L-citrulline, NO acts as a pivotal vasodilator mediator liberated from endothelial cells of ocular blood vessels. There is evidence that constant formation of NO by eNOS provides the maintenance of a basal vasodilator tone in the optic nerve head of humans and experimental animals [26–30], which is a precondition of sufficient blood supply to this tissue. Earlier studies suggested that vascular dysregulation played an important role in the etiology of glaucoma [31, 32]. In accordance with this, Polak et al. observed the perfusion of the optic nerve head during NOS inhibition and found differences in ocular blood flow response between patients with POAG and controls, indicating an abnormal NO system and NOS activity in POAG patients [33]. Further, it was reported that the increased presence of eNOS in vascular endothelia may be neuroprotective by causing vasodilatation and increased blood flow in the glaucomatous tissue [34]. Besides, the activity of NOS in trabecular meshwork was observed in patients with POAG [35]. Based on these evidences, it is reasonable to assume that the polymorphisms of eNOS are associated with the pathogenesis of POAG.

Rs2070744 and rs1799983 are the most important identified functional polymorphisms of the eNOS. The polymorphism of the promoter region of eNOS rs2070744 has been considered to be related to nonarteritic anterior ischemic optic neuropathy (NAION), coronary spasm, myocardial infarction, and coronary artery disease [36–39]. This polymorphism reduces the transcription rate of the eNOS gene and then lowers eNOS mRNA and serum nitrite/nitrate levels [40, 41]. As for eNOS rs1799983, the polymorphism has been associated with ischemic shock, coronary spasm, coronary artery disease, myocardial infarction, and NAION [38, 39, 42–44]. As this polymorphism is located in a coding region, it might be in relation to altered eNOS function and functional changes of the endothelium [45, 46]. Several investigations to date were conducted to explore the links between these two polymorphisms and POAG but achieved inconsistent conclusions. Therefore, the present meta-analysis was performed to determine whether or not these two polymorphisms could predict susceptibility to POAG.

In our study, we observed associations between eNOS gene variants and POAG, particularly among the women, revealing some sex-related facts in pathogenesis. Several lines of evidence suggest the sexually dimorphic effects of eNOS. In a series of animal studies, the expression levels of eNOS exhibited sex disparity [47] and displayed different degrees of inhibition under the sex-dependent miR-222 regulation [48]. In a human study of 373 glaucoma cases and 1082 controls, Kang et al. found that eNOS SNPs showed significant interactions with current postmenopausal hormone use in relation to high tension POAG [49]. These findings are in line with our results. Although the basis of molecular mechanisms is not clear, we believe that there are several factors that may influence this discrepancy. Concerning biological factors, circulating estrogen may act directly on eNOS through nongenomic effects, resulting in rapid dilatation of blood vessels [50, 51]. One recent study also indicates that estrogen induces NO production via NOS activation in endothelial cells [52]. Furthermore, women in most part of the world are more likely to adopt healthy lifestyle [53–56]. For example, cigarette smoking is proved to contribute to endothelial dysfunction through the uncoupling of the eNOS-mediated synthesis of NO [57, 58] and a series of studies indicate that women obtained lower tobacco consumption than their male fellows [59–61].

For our study, we have put considerable efforts and attempted to minimize every bias and gain stable and reliable results; however, there are still some limitations. Firstly, studies involved in the present meta-analysis were limited to published full-text articles in English. We failed to track the unpublished articles or ones published in other languages to obtain data for analysis, causing an influence on the completeness of the data. Secondly, although we collected and reviewed all the relevant studies, only five eligible ones were included for analysis and the sample size of the individual studies was not sufficiently large, which could increase the likelihood of type I and type II errors. As for rs2070744, we excluded one study with significant HWE deviation, further decreasing the overall sample size of our study. Therefore our results should be interpreted with caution until these findings can be replicated in other large datasets. Stratified analysis of ethnicity and sex also encountered the similar problem due to the lack of detailed data. Despite all of these limitations, we believe our study would be beneficial to a better understanding of the association between eNOS polymorphisms and POAG. Moreover, our analysis has also revealed the limitations in the current POAG genetic studies. Hence, large-scale and well-designed studies are warranted in the future. As stated, glaucoma was estimated to disproportionally affect people in Africa and thus more research needs to be conducted in the African population. Finally, since POAG is a multifactorial disease and the roles of several genes in the pathogenesis of POAG have been established, further investigations should be performed in this direction. It is possible that specific gene-gene and gene-environment interactions may alter those associations between gene polymorphisms and POAG. We expect that as more studies become available, a more accurate estimation of the relationship of eNOS with POAG will be obtained.

In summary, the current meta-analysis suggests that TT genotype and/or T-allele in rs2070744, as well as GG genotype and/or G-allele in rs1799983, was associated with decreased risk for POAG overall and in female group. To better understand the role of genetic factors in the physiopathology of this condition, further studies are needed in large, standardized, and ethnically diverse populations.

## Figures and Tables

**Table 1:** Principle characteristics of the studies included in the meta-analysis for association between eNOS rs2070744 and POAG.

First author	Cohorts	Year	Ethnicity	Genotyping	Case					Control					Control
					Size	GG	GA	AA	MAF	Size	GG	GA	AA	MAF	HWE (*p*)
Fan [16]	All	2010	Asians	NA	397	319	72	6	0.11	201	157	43	1	0.11	0.44

Kang [17]	All	2011	Caucasians	Taqman	510	236	203	71	0.34	1444	682	598	164	0.32	0.28
	Male	2011	Caucasians	Taqman	147	72	60	15	0.31	425	203	170	52	0.32	0.06
	Female	2011	Caucasians	Taqman	363	164	143	56	0.35	1019	479	428	112	0.32	0.08

Magalhães da Silva [18]	All	2012	Caucasians	Taqman	89	55	27	7	0.23	124	72	46	6	0.23	0.28
	Male	2012	Caucasians	Taqman	28	20	7	1	0.16	63	35	24	4	0.25	0.70
	Female	2012	Caucasians	Taqman	61	35	22	4	0.25	61	37	23	1	0.20	0.97

Emam [19]	All	2014	Caucasians	PCR-RFLP	160	81	59	20	0.31	110	63	37	10	0.26	0.22
	Male	2014	Caucasians	PCR-RFLP	76	41	24	11	0.11	56	32	19	5	0.11	0.19
	Female	2014	Caucasians	PCR-RFLP	84	40	35	9	0.34	54	31	18	5	0.32	0.39

NA: data not available; MAF: Minor Allele Frequency; HWE: Hardy-Weinberg equilibrium; PCR-RFLP: polymerase chain reaction-restriction fragment length polymorphism.

**Table 2:** Principle characteristics of the studies included in the meta-analysis for association between eNOS rs1799983 and POAG.

First author	Cohorts	Year	Ethnicity	Genotyping	Case					Control					Control
					Size	TT	TC	CC	MAF	Size	TT	TC	CC	MAF	HWE (*p*)
Lin [15]	All	2005	Asians	Taqman	66	55	11	0	0.08	100	84	16	0	0.08	0.38

Fan [16]	All	2010	Asians	NA	397	310	77	10	0.12	201	157	40	4	0.12	0.45

Kang [17]	All	2011	Caucasians	Taqman	518	202	232	84	0.39	1501	580	673	248	0.39	0.03
	Male	2011	Caucasians	Taqman	153	65	66	22	0.36	457	166	215	76	0.40	0.65
	Female	2011	Caucasians	Taqman	365	137	166	62	0.40	1044	414	458	172	0.38	0.02

Magalhães da Silva [18]	All	2012	Caucasians	Taqman	89	42	39	8	0.31	123	74	38	11	0.24	0.07
	Male	2012	Caucasians	Taqman	28	16	10	2	0.25	61	35	22	4	0.25	0.83
	Female	2012	Caucasians	Taqman	61	26	28	7	0.34	62	39	20	3	0.21	0.83

Emam [19]	All	2014	Caucasians	PCR-RFLP	160	63	59	38	0.42	110	60	38	12	0.28	0.12
	Male	2014	Caucasians	PCR-RFLP	76	32	26	18	0.08	56	30	19	7	0.08	0.17
	Female	2014	Caucasians	PCR-RFLP	84	31	33	20	0.12	54	30	19	5	0.12	0.44

NA: data not available; MAF: Minor Allele Frequency; HWE: Hardy-Weinberg equilibrium; PCR-RFLP: polymerase chain reaction-restriction fragment length polymorphism.

**Table 3:** Summary risk estimates for association between eNOS rs2070744 and POAG.

	Comparisons	Studies (*n*)	Model	Pooled estimate	*p*_*Z*_	Heterogeneity *I* ^2^ (%)	*p*_*Q*_	Egger's test (*p*)	Begg's test (*p*)
				OR (95% CI)					
Overall	T versus C	4	F	0.736 (0.594–0.912)	0.005	44.8	0.143	0.724	0.734
	TT versus CC	4	F	0.498 (0.296–0.838)	0.009	21.6	0.279	0.236	1.000
	TC versus CC	4	F	0.725 (0.425–1.239)	0.240	25.0	0.264	0.561	1.000
	TT + TC versus CC	4	F	0.573 (0.348–0.943)	0.029	25.1	0.263	0.379	1.000
	TT versus TC + CC	4	F	0.746 (0.575–0.967)	0.027	34.6	0.205	0.873	1.000

Female	T versus C	2	F	0.490 (0.333–0.721)	0.000	0.0	0.887		
	TT versus CC	2	F	0.268 (0.112–0.642)	0.003	0.0	0.913		
	TC versus CC	2	F	0.489 (0.200–1.196)	0.117	0.0	0.732		
	TT + TC versus CC	2	F	0.328 (0.144–0.749)	0.008	0.0	0.764		
	TT versus TC + CC	2	F	0.423 (0.260–0.689)	0.001	0.0	0.896		

Male	T versus C	2	F	0.711 (0.467–1.081)	0.111	8.4	0.296		
	TT versus CC	2	F	0.496 (0.211–1.175)	0.111	0.0	0.452		
	TC versus CC	2	F	0.604 (0.244–1.496)	0.276	0.0	0.623		
	TT + TC versus CC	2	F	0.513 (0.230–1.144)	0.103	0.0	0.476		
	TT versus TC + CC	2	F	0.688 (0.406–1.165)	0.164	0.0	0.328		

Asians	T versus C	2	F	0.971 (0.695–1.358)	0.864	0.0	0.967		
	TT versus CC	2	F	0.790 (0.244–2.558)	0.694	—	—		
	TC versus CC	2	F	0.770 (0.227–2.610)	0.675	—	—		
	TT + TC versus CC	2	F	0.786 (0.243–2.537)	0.687	—	—		
	TT versus TC + CC	2	F	0.990 (0.684–1.431)	0.955	0.0	0.921		

Caucasians	T versus C	2	F	0.607 (0.460–0.803)	0.001	3.2	0.310		
	TT versus CC	2	F	0.444 (0.249–0.791)	0.006	46.1	0.173		
	TC versus CC	2	R	0.715 (0.394–1.296)	0.269	62.4	0.103		
	TT + TC versus CC	2	R	0.534 (0.308–0.925)	0.025	57.7	0.124		
	TT versus TC + CC	2	F	0.563 (0.390–0.812)	0.002	0.0	0.813		

OR: odds ratio; CI: confidence interval; *p*_*Z*_: *p* value for *Z* test; *p*_*Q*_: *p* value for *Q*-test; F: fixed-effects mode; R: random-effects model; —: data not available.

**Table 4:** Summary risk estimates for association between eNOS rs1799983 and POAG.

	Comparisons	Studies (*n*)	Model	Pooled estimate OR (95% CI)	*p*_*Z*_	Heterogeneity *I* ^2^ (%)	*p*_*Q*_	Egger's test (*p*)	Begg's test (*p*)
Overall	G versus A	4	F	0.928 (0.817–1.053)	0.247	0.0	0.694	0.851	0.734
	GG versus AA	4	F	0.753 (0.568–0.997)	0.048	0.0	0.831	0.039	0.308
	GA versus AA	4	F	0.745 (0.559–0.993)	0.045	0.0	0.720	0.149	0.089
	GG + GA versus AA	4	F	0.752 (0.576–0.983)	0.037	0.0	0.824	0.033	0.089
	GG versus GA + AA	4	F	0.979 (0.832–1.153)	0.803	0.0	0.584	0.832	1.000

Female	G versus A	3	F	0.852 (0.724–1.003)	0.054	0.0	0.869		
	GG versus AA	3	F	0.665 (0.471–0.938)	0.020	0.0	0.651		
	GA versus AA	3	F	0.674 (0.474–0.958)	0.028	0.0	0.505		
	GG + GA versus AA	3	F	0.666 (0.481–0.923)	0.015	0.0	0.626		
	GG versus GA + AA	3	F	0.894 (0.720–1.108)	0.306	0.0	0.684		

Male	G versus A	3	F	1.067 (0.839–1.358)	0.595	20.7	0.283		
	GG versus AA	3	F	1.085 (0.641–1.836)	0.124	0.0	0.431		
	GA versus AA	3	F	1.041 (0.604–1.793)	0.885	0.0	0.557		
	GG + GA versus AA	3	F	1.066 (0.643–1.768)	0.804	0.0	0.456		
	GG versus GA + AA	3	F	1.088 (0.798–1.485)	0.593	0.0	0.378		

Asians	G versus A	1	—	1.065 (0.726–1.564)	0.746	—	—		
	GG versus AA	1	—	0.339 (0.040–2.837)	0.682	—	—		
	GA versus AA	1	—	0.279 (0.032–2.397)	0.245	—	—		
	GG + GA versus AA	1	—	0.326 (0.039–2.725)	0.301	—	—		
	GG versus GA + AA	1	—	1.146 (0.756–1.737)	0.520	—	—		

Caucasians	G versus A	3	F	0.912 (0.797–1.043)	0.180	0.0	0.641		
	GG versus AA	3	F	0.768 (0.578–1.021)	0.069	0.0	0.855		
	GA versus AA	3	F	0.765 (0.572–1.023)	0.071	0.0	0.776		
	GG + GA versus AA	3	F	0.767 (0.585–1.006)	0.055	0.0	0.866		
	GG versus GA + AA	3	F	0.952 (0.798–1.137)	0.803	0.0	0.584		

OR: odds ratio; CI: confidence interval; *p*_*Z*_: *p* value for *Z* test; *p*_*Q*_: *p* value for *Q*-test; F: fixed-effects mode; —: data not available.
